# Smoking increases oral mucosa susceptibility to *Candida albicans* infection via the Nrf2 pathway: In vitro and animal studies

**DOI:** 10.1111/jcmm.16724

**Published:** 2021-06-21

**Authors:** Pei Ye, Wei Chen, Fan Huang, Qin Liu, Ya‐Nan Zhu, Xiang Wang, Xiao‐Dong Han, Wen‐Mei Wang

**Affiliations:** ^1^ Nanjing Stomatological Hospital & State Key Laboratory of Analytical Chemistry for Life Science Medical School Nanjing University Nanjing China; ^2^ Jiangsu Key Laboratory of Molecular Medicine Nanjing University Nanjing China

**Keywords:** *Candida albicans*, NLRP3 inflammasome, Nrf2, oral immunity, oxidative stress, smoking

## Abstract

Smoking and *Candida* *albicans* (*C*. *albicans*) infection are risk factors for many oral diseases. Several studies have reported a close relationship between smoking and the occurrence of *C*. *albicans* infection. However, the exact underlying mechanism of this relationship remains unclear. We established a rat infection model and a *C. albicans*‐Leuk1 epithelial cell co‐culture model with and without smoke exposure to investigate the mechanism by which smoking contributes to *C*. *albicans* infection. Oral mucosa samples from healthy individuals and patients with oral leucoplakia were also analysed according to their smoking status. Our results indicated that smoking induced oxidative stress and redox dysfunction in the oral mucosa. Smoking‐induced Nrf2 negatively regulated the NLRP3 inflammasome, impaired the oral mucosal defence response and increased the oral mucosa susceptibility to *C*. *albicans*. The results suggest that the Nrf2 pathway could be involved in the pathogenesis of oral diseases by mediating an antioxidative response to cigarette smoke exposure and suppressing host immunity against *C*. *albicans*.

## 1 INTRODUCTION

*Candida albicans*, also known as *C*. *albicans*, is a commensal fungus widely present in different parts of the human body, such as the skin, oral cavity, and gastrointestinal and urogenital tracts.^1^ *Candida* *albicans* can cause superficial or deep organ infections in immunosuppressed patients. In healthy hosts, oral epithelial cells provide the first line of defence against *C*. *albicans*. However, localized epithelial alterations can damage the mucosal barrier and cause Candida infections. Many epidemiological studies have reported a close relationship between smoking and the occurrence of *C*. *albicans* infection.^2^, ^3^ A previous study reported that patients with clinically suspected oral leucoplakia (OLK) who had smoking habit were significantly associated with Candida infection.^4^ We have previously shown that smoking exacerbates oral *C*. *albicans* infection and dampens the defence response of epithelial cells in rats.^5^

Cigarette smoke (CS) contains more than 7000 known molecules,^6^ including a complex mixture of oxidants and various free radicals. It can change the oral environment and cause significant oxidative stress, which in turn damages the oral epithelial barrier. Previous studies have revealed that the body is sensitive to external stimuli and is prone to infection when its redox state is out of balance.^7^ Reactive oxygen species (ROS) generated by CS can damage the structure and function of infected cells. Several oxidative stress–sensing proteins in the epithelial cells, including nuclear factor erythrocyte 2–related factor 2 (Nrf2), are activated upon exposure to smoking.^8^ In the presence of extraneous electrophilic chemicals, Nrf2 is transmitted to the nucleus and increases the expression of antioxidant enzyme genes, such as NAD(P)H dehydrogenase quinone‐1 (*NQO‐1*) and haem‐oxygenase‐1 (*HO‐1*). Moreover, Nrf2 activation is generally considered to have anti‐inflammatory effects.^9^, ^10^ It has been reported that Nrf2 reduces the lipopolysaccharide (LPS)‐induced secretion of pro‐inflammatory cytokines, including interleukin (IL)‐6 and IL‐1β.^11^ Meanwhile, the crosstalk between Nrf2 and NOD‐like receptor family pyrin domain containing 3 (NLRP3) inflammasome has recently received much attention.^12^ The NLRP3 inflammasome recognizes several pathogens and plays an important role in opposing *C*. *albicans* infection.^13^

The exact mechanism by which smoking increases susceptibility to *C*. *albicans* infection has not yet been established. Previous studies mainly have focused on the influence of smoking on the virulence of *C*. *albicans* in vitro and have paid little attention to the in vivo immune dysfunction caused by smoking.^14^, ^15^ Thus, we believe that the Nrf2 signalling pathway may be involved regulating smoking‐induced *C*. *albicans* infection. In this study, we established in vivo and in vitro smoking and *C*. *albicans* interaction models to investigate the mechanism linking epithelial infection and smoking.

## 2 MATERIALS AND METHODS

### 2.1 Animals and treatments

Wistar rats (145‐155 g) aged 5 weeks were used in this study in 1:1 sex ratio. The rats were housed under standard conditions with a 12‐hours/12‐hours light/dark cycle. The rats were randomly divided into four groups (n = 8 per group): control, CS, *C*. *albicans* and CS + *C. albicans*. The rats were administered CS or infected with *C*. *albicans* for 24 weeks as described previously.^5^ Each rat was exposed to the smoke from two cigarettes twice daily for 0.5 hour in each session. For *C*. *albicans* infection, a sterile and uniform‐sized cotton packing saturated with 100 μL of a 1.0 × 10^8^ CFU/mL *C*. *albicans* suspension in PBS was placed in the oral cavity for approximately 4‐6 hours, while the animals remained sedated with 2% pentobarbital sodium. The rats were inoculated once per week until 1 week before execution. The rats in the CS + *C. albicans* group had already been exposed to CS for 2 weeks before *C*. *albicans* infection. After 24 weeks of treatment, the rats were anaesthetized and killed. Oral tissues and serum were collected for oxidative biomarker measurements and biochemical analysis. The experimental procedures were carried out in accordance with the Guide for the Welfare and Use of Laboratory Animals and approved by the local Ethics Committee (IRB Approval Number: 2015NL‐001 (ks)).

### 2.2 Cells and strains

Leuk1, an immortalized human oral mucosa epithelial cell line, was cultured in dishes containing defined keratinocyte serum‐free medium (K‐SFM; GIBCO, Invitrogen, Grand Island, USA) at 37°C, 5% CO_2_. The *C*. *albicans* strain 62342 was cultured at 30°C in yeast peptone dextrose (YPD) medium. *Candida* *albicans* was collected and washed twice with sterile PBS; then, it was suspended, counted and co‐incubated with Leuk1 cells at a ratio of 1:4 for 12 hours.

### 2.3 Treatment with cigarette smoking extract (CSE)

Cigarette smoking extract was prepared as described previously.^16^, ^17^ Smoke from a 3R4F cigarette (Kentucky Tobacco Research Institute, Lexington, USA) was drawn into 50 mL of cell culture medium (K‐SFM) using a peristaltic pump (LongerPumper). The prepared filtered solution was considered to be 100% CSE. A 4% CSE concentration was used as the working concentration in this study as it did not influence epithelial viability. Leuk1 cells (1 × 10^5^/mL) in the logarithmic growth phase were seeded in 6‐well plates, and 4% CSE was added to the wells for 72 hours after the cells grew and adhered to the well wall.

### 2.4 Sulphoraphane (SFN) treatment and lentivirus transfection of Nrf2

Sulphoraphane is a natural antioxidant agent present in cruciferous vegetables that activates Nrf2.^18^ SFN (5 μmol/L; Sigma‐Aldrich) was used in this study. A certain concentration of LV‐Nrf2(‐CON) (GeneChem Co.) was added to Leuk1 cells at an MOI of 50.

### 2.5 Periodic acid–Schiff (PAS) staining and fungal burdens of the tissues

The oral mucosa was stained with PAS using an appropriate method.^19^ Rat tongues were collected and homogenized in sterile PBS. Ten‐fold dilutions of the homogenate were incubated for 48 hours at 30°C, and fungal colonies were counted.^20^

### 2.6 Immunohistochemical examination (IHC) and immunofluorescence microscopy

Immunohistochemical examination analysis of oral mucosa tissues was performed as previously described.^21^ The following primary antibodies were used: Nrf2, HO‐1, NQO‐1, p‐Nrf2, NLRP3, IL‐1β and IL‐18 (Abcam). The immunostained sections were examined under an optical microscope (Olympus). The IHC results were scored as follows: (a) percentage of positive cells: 0 (0%), 1 (<10%), 2 (10%‐50%), 3 (51%‐80%) and 4 (>80%) and (b) intensitys: 0 (negative staining), 1 (mild reaction), 2 (moderate reaction) and 3 (intense reaction). The final score for protein expression, which ranged from 0 to 12, was calculated by multiplying the scores for the positive cell percentage and the staining intensity.^22^ Immunostaining was performed to detect the expression of Nrf2 axis in epithelial cells. Cy3‐conjugated goat anti‐rabbit antibody (Invitrogen) was used as the secondary antibody. The nuclei of each sample were counterstained with 4′,6‐diamidino‐2‐phenylindole (DAPI; Millipore Sigma), and the images were obtained using under a laser scanning confocal microscope (Olympus).

### 2.7 Measurement of malondialdehyde (MDA) levels, superoxide dismutase (SOD) activity and reduced glutathione (GSH)/oxidized glutathione (GSSG) ratio

Rat serum and Leuk1 cells were collected and used for analysis. The MDA level, the SOD activity and the GSH/GSSH ratio were measured using commercial kits (Beyotime) following the manufacturer's instructions. The MDA content and SOD activity of the cells were normalized to the protein levels.

### 2.8 Detection of ROS

A reactive oxygen species assay kit (Beyotime) was used to detect the accumulation of ROS in Leuk1 cells. The cells were collected and diluted in serum‐free 2′,7′‐dichlorodihydrofluorescein diacetate (DCFH‐DA) medium in the dark at 37°C for 20 minutes. Fluorescence intensities before and after stimulation were determined by confocal microscopy or using a fluorescence microplate system in real time.

### 2.9 RNA isolation and quantitative polymerase chain reaction (Q‐PCR)

RNA was extracted and transcribed into cDNA which was then amplified using a SYBR Green Q‐PCR kit (Roche) on a 7300 Real‐Time PCR System (Applied Biosystems). Relative mRNA levels were quantified using the 2^−△△Ct^ relative expression method. GAPDH was selected as the reference gene for this study. The specific primers for the corresponding genes are provided in Supplemental File.

### 2.10 Western blotting

Proteins were extracted from the Leuk1 cells, and cellular lysates were analysed as previously described.^23^ The primary antibodies used were mouse anti‐NLRP3, rabbit anti‐caspase‐1, Nrf2, HO‐1, NQO‐1 and GAPDH (all from Abcam). HRP‐conjugated goat anti‐mouse/rabbit immunoglobulin G (Boster) was used as the secondary antibody. Protein levels were quantified by densitometry using Image‐Pro Plus and normalized to the GAPDH level.

### 2.11 Enzyme‐linked immunosorbent assay (ELISA)

Rat serum samples and cell culture supernatants were used to determine the IL‐6, TNF‐α, IL‐1β and IL‐18 concentrations using ELISA kits (MultiSciences Biotech Co.) according to the manufacturer's instructions.

### 2.12 Study patients

The study was approved by the Ethics Committee of the local organization (IRB approval number: 2015NL‐001(ks)). All patients were informed about the study and provided written informed consent. From 2015 to 2018, 30 patients with OLK were enrolled from the Department of Oral Medicine in our hospital, and their diagnoses were confirmed using appropriate pathology tests. Additionally, 16 patients who underwent orthognathic surgery or impacted teeth extraction were selected as normal controls (using pathology tests, all of them were confirmed to have no malignant changes). They were equally divided into two groups based on their smoking habit. Those who reported the use of more than 10 cigarettes per day were classified as smokers.^24^ Considering their health, the smokers were encouraged to cease smoking and receive regular periodontal treatment. The oral mucosa from the selected subjects was examined by cotton swab sampling. The strains were identified by comparing DNA sequence data for the internal transcribed spacer, and the relative abundance of *C*. *albicans* was determined. A biopsy tissue was collected from the surgical site in the oral cavity of each patient for IHC analysis.

### 2.13 Statistical analysis

The experimental results are expressed as the mean ± SD. The results were analysed using two‐way analysis of variance followed by a least significant difference test as a post hoc analysis among groups, or the two‐tailed unpaired Student's *t* test between two groups. SPSS 19.0 and GraphPad Prism were used to process the data. The results were considered statistically significant at *P* < .05.

## 3 RESULTS

### 3.1 Smoking increased the proliferation of *C*. *albicans*

**FIGURE 1:**
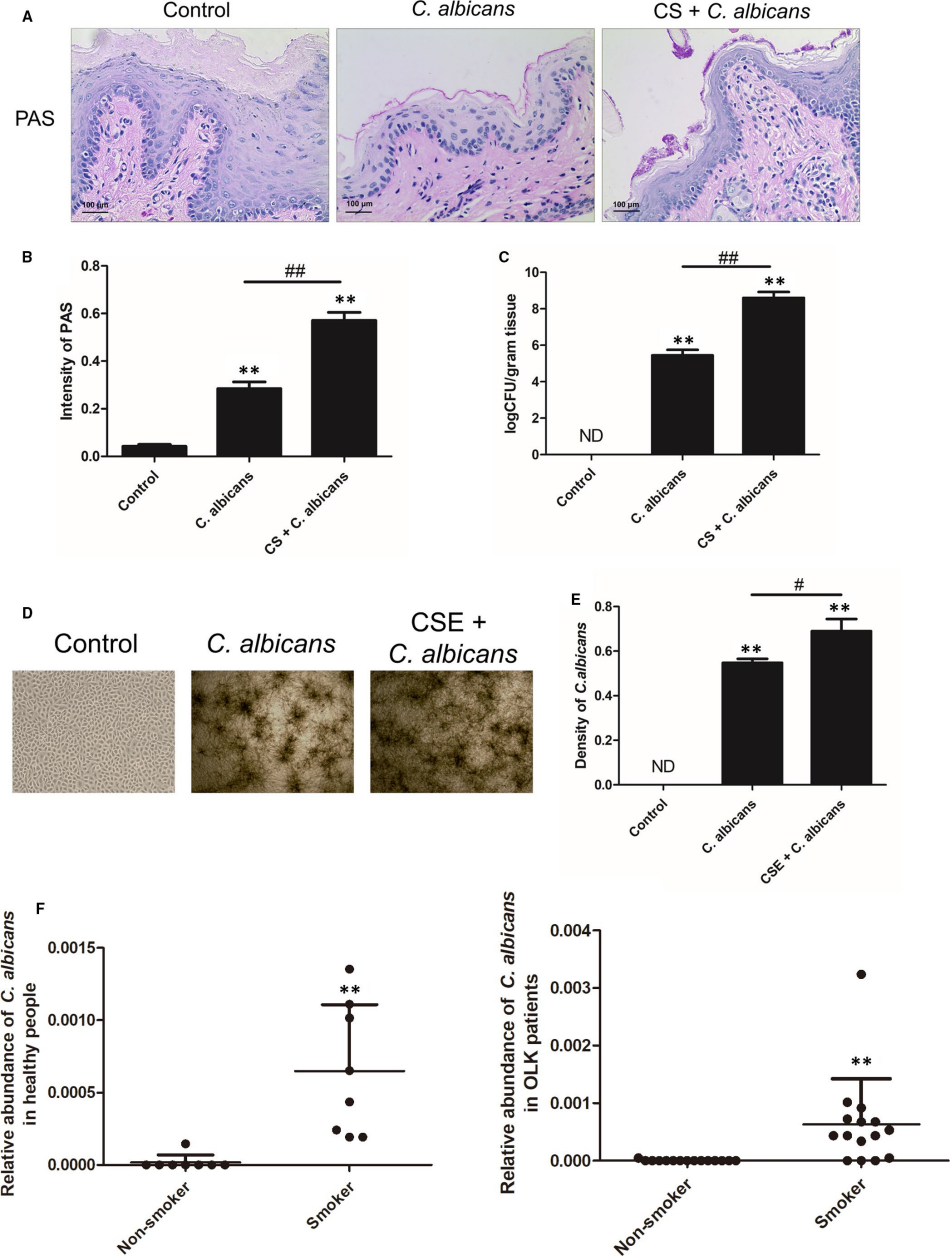
Smoking increased *Candida* *albicans* proliferation in rats, Leuk1 cells and patients. A, Examples of PAS staining of oral tissue after 6 mo of treatment. CS + *C. albicans* group showed more budding cells and pseudohyphae of Candida on the surface of buccal mucosa (original magnification: 200×, scale bar: 100 μm). B, Semiquantitative analysis of PAS staining. The degree of *C*. *albicans* infection in PAS‐stained tissues was assessed. The co‐exposed group was more susceptible to *C*. *albicans*. C, Quantitative fungal burden on tongues in the rat model. A significantly increased fungal burden was observed in the co‐exposed group compared to that in *C*. *albicans* group. D, Microscopic view of *C*. *albicans* incubated with Leuk1 cells for 24 h (original magnification, 40×). E, Semiquantitative analysis of *C*. *albicans* colonies. *Candida* *albicans* colonization intensified after CSE treatment. F, The presence of *C*. *albicans* in saliva of human subjects is shown. Each bar represents the mean ± SD. **P < .01, compared with control. ^#^ *P* < .05, ^##^ *P* < .01, compared between two groups. ND:none detected. PAS: Periodic acid–Schiff; CS: cigarette smoke; CSE: cigarette smoke extract; *C*. *albicans*: *Candida albicans*

Periodic acid–Schiff staining revealed budding cells and pseudohyphae of Candida on the surface of the buccal mucosa, as shown in Figure 1A. However, the staining intensity in the CS‐treated rats was significantly higher than that in the non‐CS‐treated rats (*P* < .01; Figure 1B), indicating that the CS‐exposed group was susceptible to *C*. *albicans* infection. In addition, we noticed that the *C*. *albicans* burden on rat tongue in the CS + *C. albicans* group increased remarkably compared to that in the *C*. *albicans* group (*P* < .01, Figure 1C). We then observed the density of in vitro colonies formed from infected oral epithelial cells under a microscope (Figure 1D). *Candida* *albicans* colonization intensified after CSE treatment (*P* < .05; Figure 1E). The *C*. *albicans*‐positive cases reported from both healthy people and patients with OLK were more common in smokers than in non‐smokers (*P* < .01; Figure 1F). These results indicate that smoking promoted the growth of Candida cells.

### 3.2 Biomarkers of oxidative injury in oral mucosa fungal infection

**FIGURE 2:**
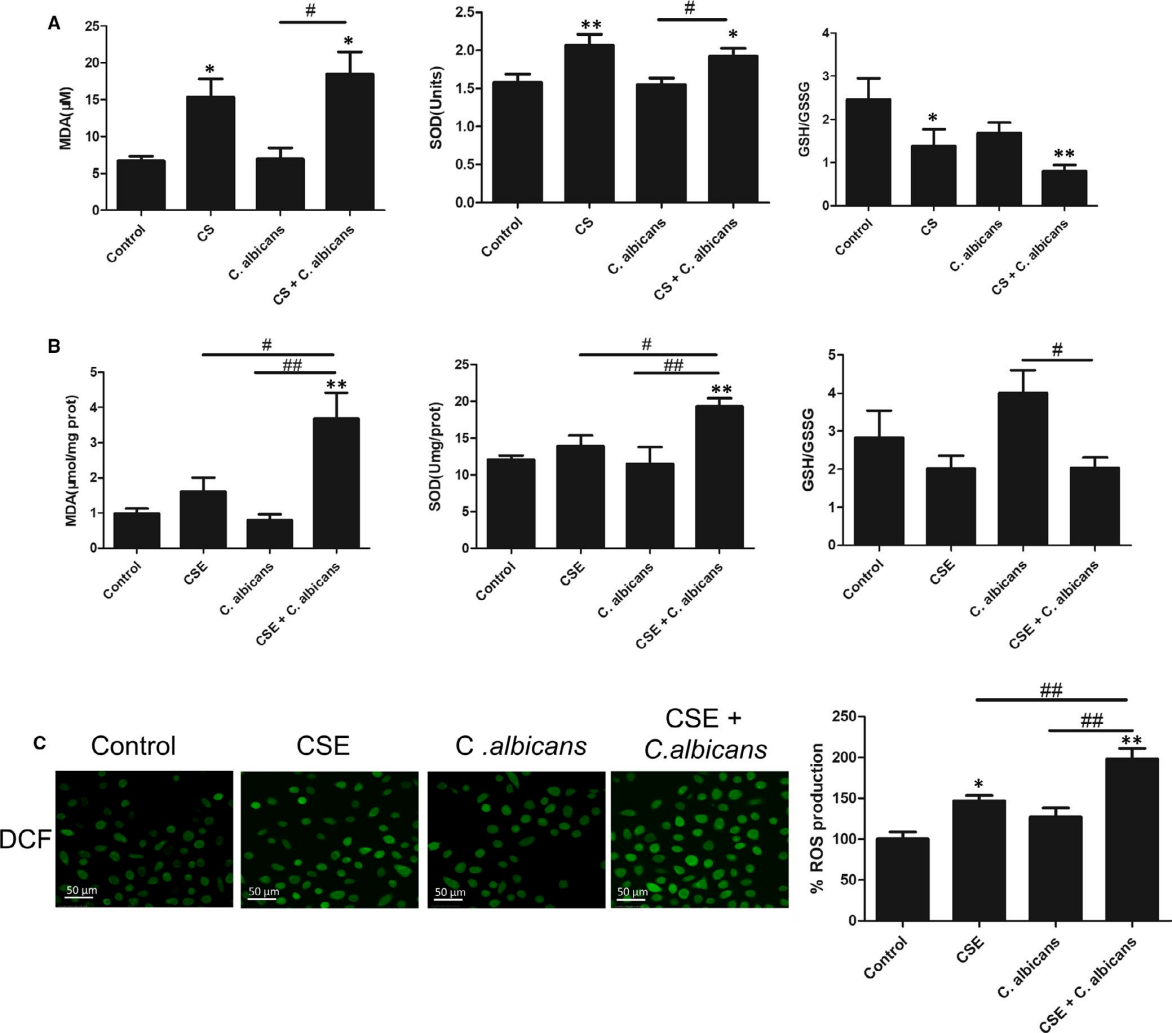
Biomarkers of oxidative injury in oral mucosa fungal infection. A, MDA level, SOD activity and GSH/GSSG ratio in the serum of the rat model were detected by commercial kits. B, The corresponding indexes in Leuk1 cells were also measured. The MDA levels and SOD activity increased, while GSH/GSSG levels decreased after CS or CSE treatment in vivo and in vitro. C, Smoking induces ROS production in Leuk1 cells. Left: representative micrographs show that CSE treatment increases ROS production, as shown by DCF (in green; original magnification: 400×, scale bar: 50 μm). Right: fluorescence intensity measured using a fluorescence microplate system. Each bar represents the mean ± SD. *P < .05, **P < .01, compared with control. ^#^ *P* < .05, ^##^ *P* < .01, compared with the *Candida* *albicans* group. CS: cigarette smoke; CSE: cigarette smoke extract; *C*. *albicans*: *Candida albicans*; ROS: reactive oxygen species; MDA: malondialdehyde; SOD: superoxide dismutase; GSH/GSSG: reduced glutathione/oxidized glutathione; DCF: 2′,7′‐dichlorofluorescein

We found that MDA content and SOD activity increased after CS or CSE treatment, respectively, in infected rats or cells, while GSH/GSSG levels decreased in vitro (Figure 2A,B). The ROS levels in co‐incubated cells with or without CSE were investigated; the fluorescence intensity did not change significantly in *C*. *albicans*‐infected cells. However, the results demonstrated marked ROS accumulation in CSE‐exposed co‐cultured cells compared to unexposed co‐cultured cells (*P* < .01; Figure 2C).

### 3.3 Nrf2 was activated in experimental oral infection and *C*. *albicans*‐exposed Leuk1 cells

**FIGURE 3:**
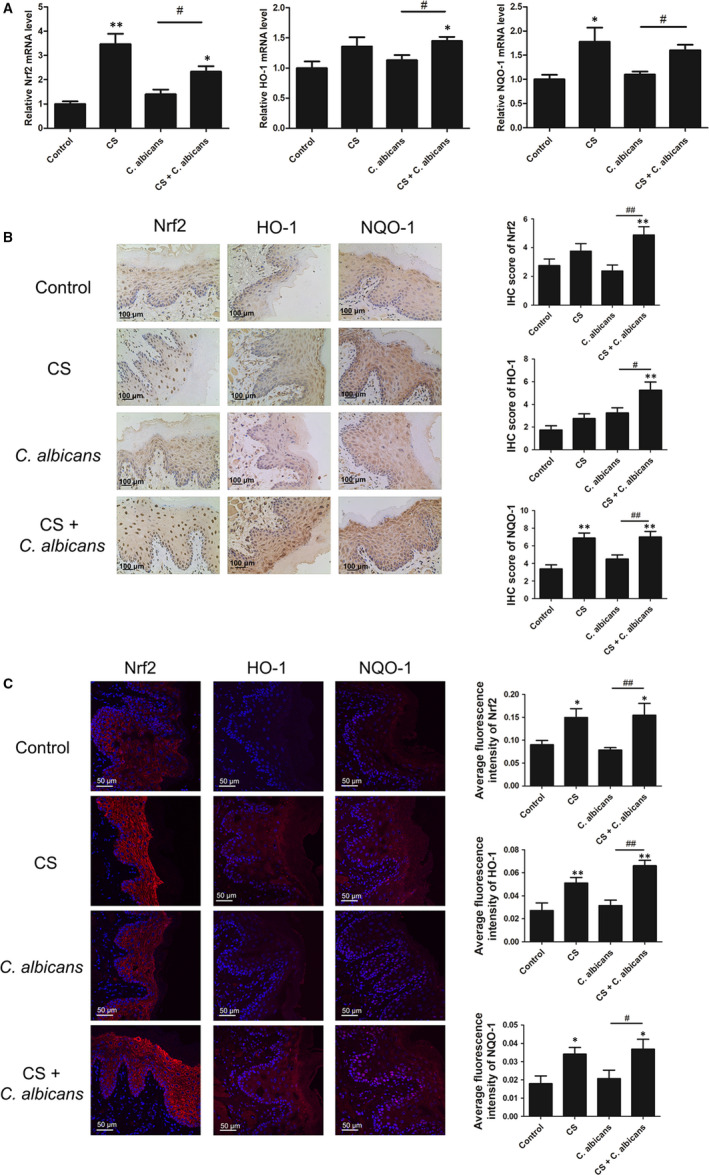
The mRNA and protein expression levels of genes in the Nrf2 pathway were measured in the rat model. A, Q‐PCR assay of the Nrf2 pathway. B, IHC staining in the rat model. Left: representative photomicrographs of Nrf2, HO‐1 and NQO‐1 (original magnification: 200×, scale bar: 100 μm). Right: histogram of IHC score from experimental tissues. C, Confocal microscopy analysis of the Nrf2 pathway. Left: merged images were obtained using a confocal microscope and analysed. The fluorescence images were captured with the red channel at 566 nm with excitation at 554 nm. Nuclei were stained blue. Sections are representative of eight rats per group (original magnification: 400× for staining, scale bar: 50 μm). Right: average fluorescence intensity. Results showed that the expression levels of the Nrf2 pathway were elevated in the CS‐exposed rats. Each bar represents the mean ± SD. *P < .05, **P < .01, compared with control. ^#^ *P* < .05, ^##^ *P* < .01, compared with the *Candida* *albicans* group. Q‐PCR: quantitative polymerase chain reaction; IHC: immunohistochemical staining; CS: cigarette smoke; *C*. *albicans*: *Candida albicans*

**FIGURE 4:**
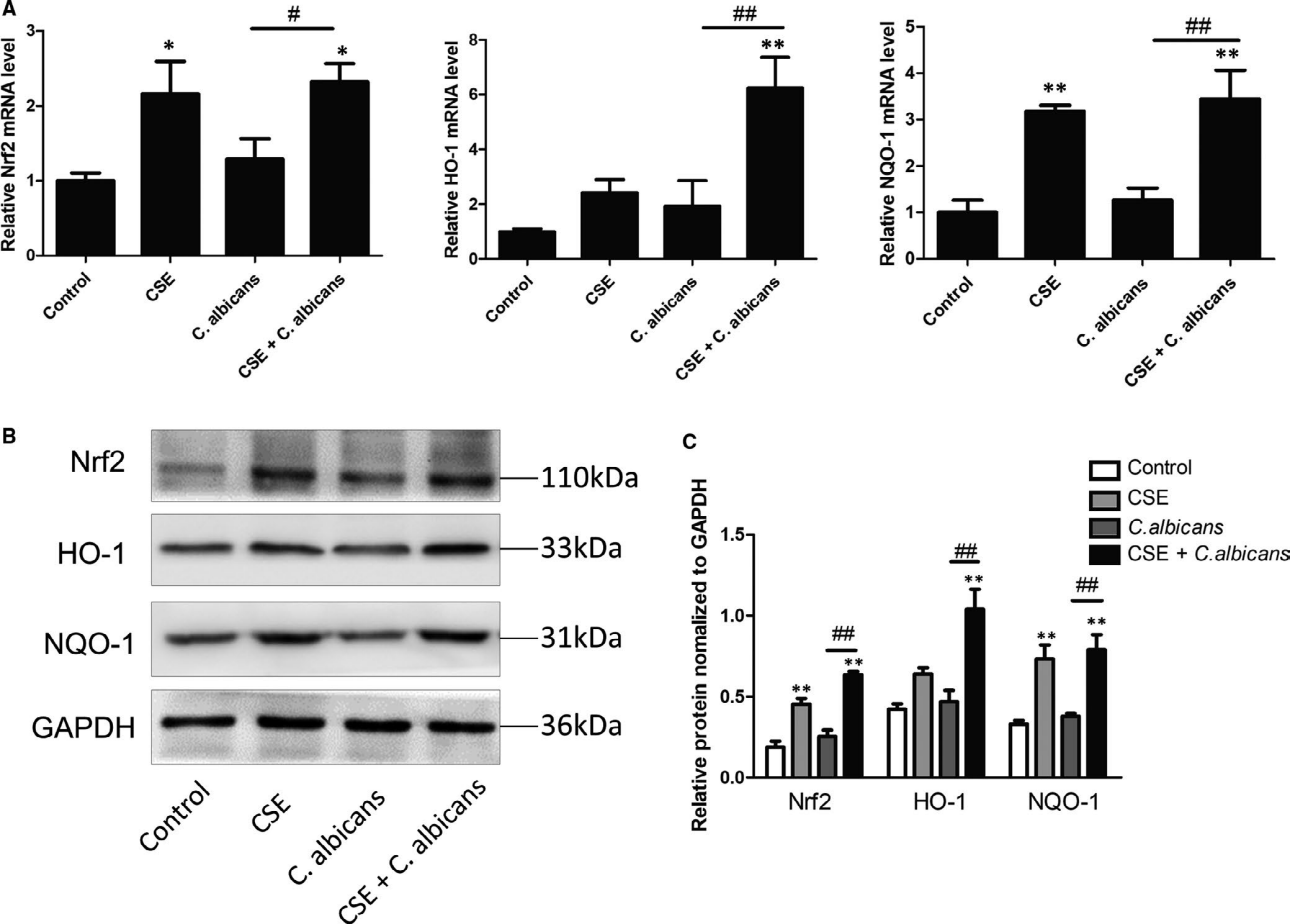
mRNA and protein expression levels of genes in the Nrf2 pathway were measured in Leuk1 cells. A, Q‐PCR assay of the Nrf2 pathway. B, Nrf2, HO‐1 and NQO‐1 expression levels were assessed by Western blotting. C, Densitometry of Western blots. GAPDH was used as a loading control. Graphs show the densitometric analysis (protein/GAPDH) of each band from three independent experiments. Results showed that the Nrf2 pathway was activated after CSE treatment. Each bar represents the mean ± SD. *P < .05, **P < .01, compared with control. ^#^ *P* < .05, ^##^ *P* < .01, compared with the *Candida* *albicans* group. CSE: cigarette smoke extract; *C*. *albicans*: *Candida albicans*

Since Nrf2 pathway regulates the cellular oxidative stress responses, we attempted to clarify whether this pathway is involved in this process. Nrf2 activation was evaluated both in an animal model and in co‐cultured cells in vitro. Q‐PCR analysis of Nrf2, HO‐1 and NQO‐1 mRNA levels showed a notable increase in the CS + *C. albicans* group compared with those in the *C*. *albicans* group (*P* < .05; Figure 3A). IHC was performed on sections of the oral mucosa of the rats. Nrf2 was expressed in both cytoplasm and nucleus but was mainly expressed in the nucleus after CS treatment. The results showed that the CS group had a higher positive ratio and stained areas for Nrf2, NQO‐1 and HO‐1 (Figure 3B). In addition, we observed similar increases in smoke‐treated rats compared to those in untreated rats using immunofluorescence (Figure 3C). Accordingly, Nrf2 activation was confirmed in the co‐cultured cell model (Figure 4). These findings indicate that smoking can activate the Nrf2 pathway in oral epithelial cells.

### 3.4 Nrf2 was involved in regulating cellular oxidation levels

**FIGURE 5:**
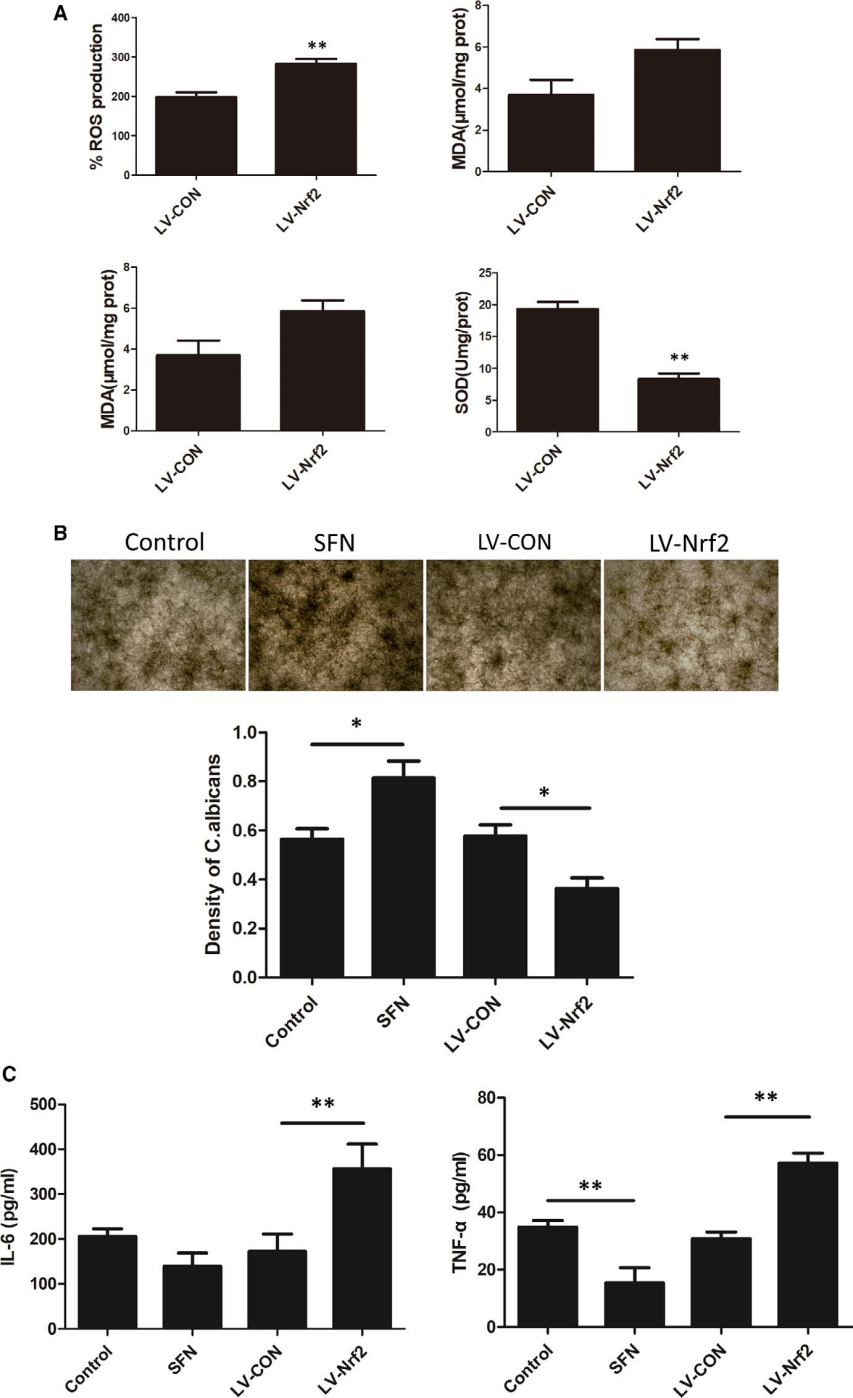
Nrf2 is involved in the regulation of cellular oxidation levels and attenuated oral epithelial antifungal immunity in the CSE‐treated *C*. *albicans* and Leuk1 co‐culture model. A, ROS fluorescence, MDA content, SOD activity and GSH/GSSG ratio were detected when Nrf2 was silenced in CSE‐treated co‐cultured cells. B, Microscopic view and semiquantitative analysis of *Candida* *albicans* colonies in different groups. C, Cytokine (IL‐6 and TNF‐α) levels in the supernatant were measured using ELISA kits. Each bar represents the mean ± SD. *P < .05, **P < .01, compared between two groups. CSE: cigarette smoke extract; *C*. *albicans*: *Candida albicans*; control: CSE‐treated *C*. *albicans* and Leuk1 co‐culture group; ROS: reactive oxygen species; MDA: malondialdehyde; SOD: superoxide dismutase; GSH/GSSG: reduced glutathione/oxidized glutathione; SFN: sulphoraphane

The increase in ROS fluorescence in transfected cells was statistically different from that in control cells. Treatment with LV‐Nrf2 showed a tendency to increase MDA levels; however, the change was not statistically significant. SOD and GSH/GSSG levels were significantly decreased. Thus, our data (Figure 5A) indicated that Nrf2 alleviates smoking‐induced oxidative stress.

### 3.5 Nrf2 attenuated oral epithelial antifungal immunity in the co‐culture model

*Candida albicans* was incubated with CSE‐treated Leuk1 cells for 24 hours in different treatment groups, and the number of colonies was determined. SFN and LV‐Nrf2 effectively regulated the Nrf2 expression (Supplemental File). *Candida albicans* growth in SFN‐treated cells showed an upward trend, while the LV‐Nrf2 group had fewer colonies (*P* < .05; Figure 5B). The results of this study confirmed that silencing Nrf2 can slow down *C*. *albicans* proliferation. We further explored the secretion of some defence factors. IL‐6 and TNF‐α levels in the LV‐Nrf2 group were significantly higher than those in the control group (*P* < .01); SFN treatment decreased the TNF‐α level (*P* < .01; Figure 5C). These results suggest that Nrf2 attenuates oral epithelial antifungal immunity.

### 3.6 Nrf2 inhibited NLRP3 inflammasome activation in the co‐culture model

We have previously reported the importance of the NLRP3 inflammasome in CS‐induced oral injury, which results in increased susceptibility to *C*. *albicans* infection.^5^ Moreover, Nrf2 has an anti‐inflammatory activity and a complex interaction network exists between the NLRP3 inflammasome and Nrf2.^25^ Since the Nrf2 pathway regulates NLRP3 and IL‐1β expression, we used lentivirus transfection and treatment with an Nrf2 agonist to investigate the link between these proteins.

**FIGURE 6:**
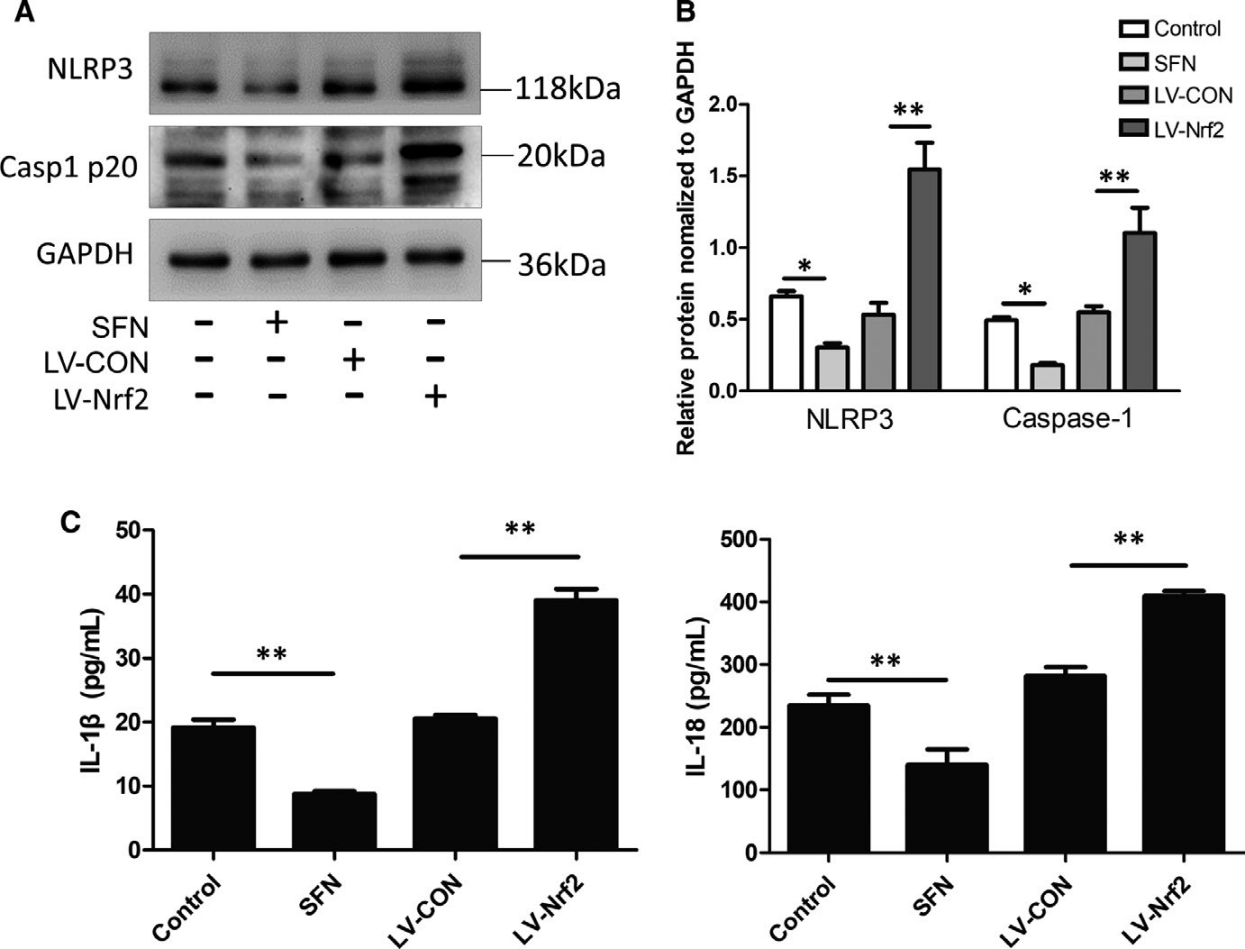
Nrf2 inhibits NLRP3 inflammasome activation in the *C*. *albicans* and Leuk1 co‐culture model. Nrf2 was silenced (LV‐Nrf2 group) or activated (SFN group) in CSE‐treated co‐cultured cells. A, NLRP3 and caspase‐12 expression levels were assessed by Western blotting. B, Densitometry of Western blots. GAPDH was used as a loading control. Graphs show the densitometric analysis (protein/GAPDH) of each band. C, Cytokine levels (IL‐β and IL‐18) in supernatant were measured using ELISA kits. N = 3. Each bar represents the mean ± SD. *P < .05, **P < .01, compared between two groups. CSE: cigarette smoke extract; *Candida* *albicans*: *Candida albicans*; control: CSE‐treated *C*. *albicans* and Leuk1 co‐culture group; SFN: sulphoraphane; Casp1 p20: active form of caspase‐12

The expression levels of NLRP3 inflammasome components were significantly reduced in SFN‐pre‐treated‐infected Leuk1 cells. In contrast, knockdown of Nrf2 in Leuk1 cells significantly increased NLRP3 inflammasome activation, whereas transfection of Leuk1 cells with a negative control virus had no effect (Figure 6A,B). The secretion of IL‐1β and IL‐18 decreased in the SFN‐treated group and increased in the LV‐Nrf2 group as demonstrated by ELISA (*P* < .01; Figure 6C). These results indicate that CSE‐induced Nrf2 activation negatively regulates the NLRP3 inflammasome.

### 3.7 Levels of the Nrf2 and NLRP3 pathways components were altered in the oral mucosa of smokers

**FIGURE 7:**
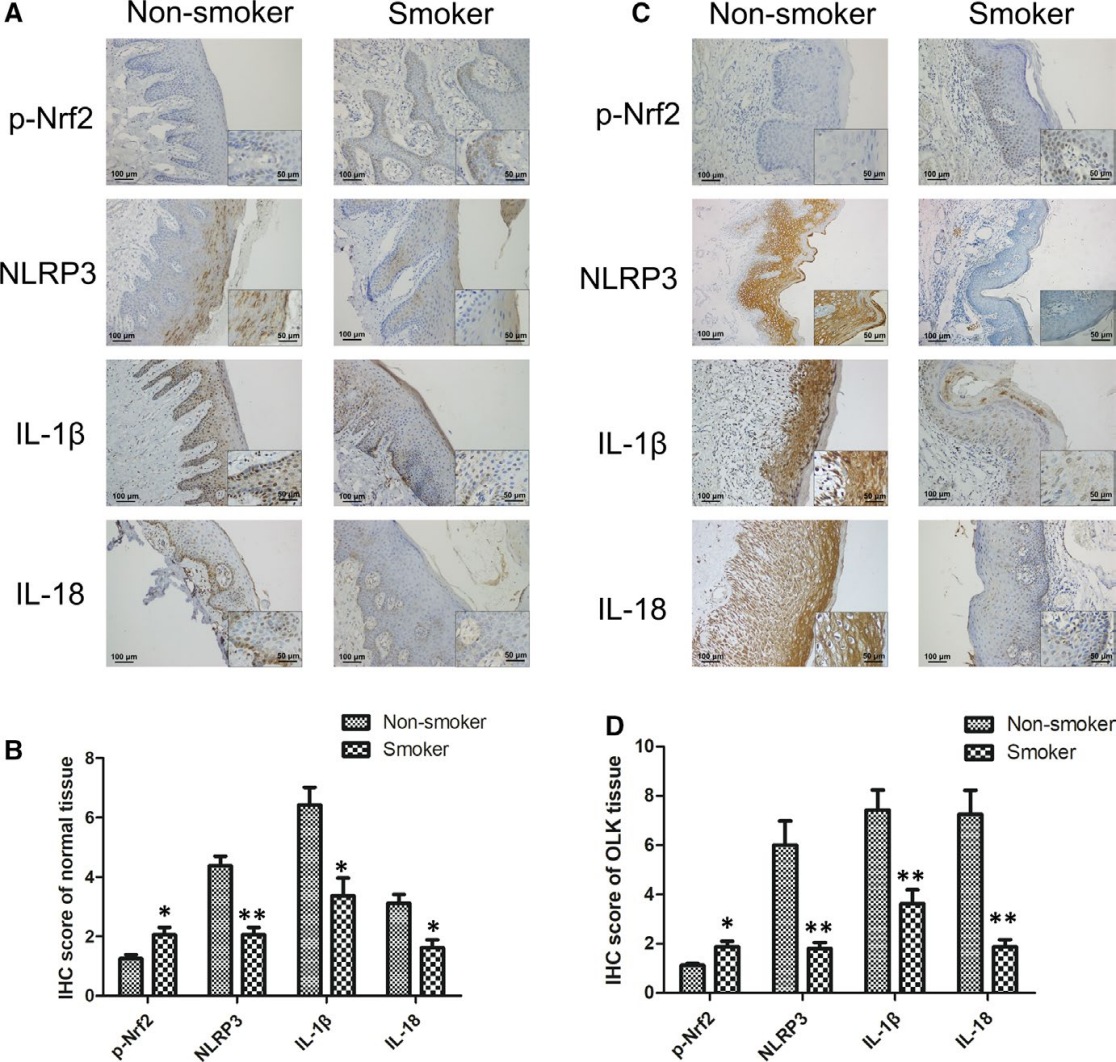
Expression of crucial molecules in the Nrf2 and NLRP3 signalling pathways was remarkably altered in the oral mucosa epithelium of smokers. A, Representative IHC staining of normal oral mucosa epithelium of non‐smokers and smokers. At high magnification, scale bar = 50 μm (insets). B, Histogram of IHC score of healthy individuals (n = 8/group). C, Representative IHC staining in non‐smokers and smokers with OLK. At power magnification, scale bar = 50 μm (insets). (D) Histogram of IHC score of patients with OLK (n = 15/group). Each bar represents the mean ± SD. *P < .05, **P < .01, compared between two groups. *C*. *albicans: Candida albicans*; IHC: immunohistochemical staining; OLK: oral leucoplakia; p‐Nrf2: phosphorylated Nrf2

Phosphorylated Nrf2 (p‐Nrf2) in the healthy non‐smoker group showed no noticeable staining, whereas the Nrf2 nuclear translocation was enhanced in smokers (Figure 7A,B). Additionally, the staining intensity of the NLRP3 pathway in normal oral mucosal tissues of healthy smokers was significantly lower than that in non‐smokers. Similarly, the intensities of p‐Nrf2, NLRP3, IL‐1β and IL‐18 staining in the oral mucosa of smokers with OLK underwent a corresponding change in the oral mucosa of smokers (*P* < .01; Figure 7C,D), indicating that smoking significantly promotes Nrf2 translocation into the nucleus and inhibits NLRP3, IL‐1β and IL‐18 expression in both healthy individuals and patients with OLK.

## 4 DISCUSSION

Smoking and *C*. *albicans* infection are risk factors for many oral diseases, such as oral candidiasis, periodontal disease and OLK.^26^, ^27^, ^28^ There is a positive correlation between smoking exposure, pack‐years and Candida colonization. However, the mechanisms by which smoking affects *C*. *albicans* proliferation are not yet understood. In this study, we propose that smoking may cause localized epithelial changes, such as influencing innate immunity, thus expediting Candida colonization. The Nrf2 system is involved in many physiological and pathological processes through the mediation of the antioxidative response and the regulation of the host inflammatory response.^29^, ^30^ However, previous studies have not identified the role of the Nrf2 pathway in smoking‐aggravated *C*. *albicans* infection.

As CS contains high levels of free radicals, we measured some redox indexes to investigate the role of Nrf2 in intracellular homeostasis. Our results indicate that smoking induces oxidative stress through ROS overgeneration. The MDA level was significantly increased, and GSH/GSSG ratio was reduced in serum and cells following exposure to smoke. In addition, the antioxidant enzymes SOD, HO‐1 and NQO‐1 were involved in tobacco‐induced oral injury. Although *C*. *albicans* infection did not alter the oral epithelium redox state, our data demonstrated that Leuk1 cells co‐cultured with *C*. *albicans* did not activate the Nrf2 pathway more than mono‐cultural epithelial cells. However, a previous study has reported that *C*. *albicans* infection significantly decreased SOD, Nrf2 and HO‐1 mRNA expression in *C*. *albicans*‐infected 293T renal epithelial cells.^31^ This discrepancy may be due to the difference in fungal strains and exposure periods. Furthermore, our results indicate that smoking triggered Nrf2 nuclear accumulation and increased the mRNA and protein expression of Nrf2 and downstream molecules both in vivo and in vitro. Taken together, these results suggest that smoking activates Nrf2, which acts as a transcription factor to maintain redox homeostasis.

Studies using Nrf2^−/−^ mice have demonstrated that Nrf2 has both protective and harmful effects on host immunity against pathogens, including bacteria and viruses.^32^, ^33^, ^34^ As reported in this study, Nrf2 attenuates oral mucosa antifungal immunity. In fungal incubation experiments, *C*. *albicans* growth was suppressed in Nrf2 knockdown cell supernatant. Furthermore, the absence of Nrf2 led to increased secretion of IL‐6, TNF‐α, IL‐1β and IL‐18, suggesting that silencing of Nrf2 can improve *C*. *albicans* clearance. A study on the defence mechanisms of the nasal and bronchial epithelium has shown that Nrf2 activation inhibits the epithelial antiviral response.^35^ Similar to the above findings, this study confirms that Nrf2 knockdown has a protective effect on smoking‐induced *C*. *albicans* infection.

Our previous study indicated that the NLRP3 inflammasome is involved in smoking‐induced oral mucosa immunosuppression.^5^ NLRP3 is a key pattern recognition receptor for *C*. *albicans*. Once activated, it activates Caspase‐1 and promotes IL‐1β and IL‐18 maturation. In mouse models of oral or vaginal *C*. *albicans* infection, NLRP3 was found to play an important role in the activation of IL‐1β and IL‐18 and elimination of mucosal infection.^36^, ^37^

Several studies have suggested an interaction between Nrf2 and NLRP3.^38^, ^39^, ^40^ However, whether there is an association between Nrf2 and NLRP3 under smoking conditions has not been clarified. In this animal model, up‐regulated Nrf2 and down‐regulated NLRP3 inflammasome are both involved in the pathogenesis of smoking‐induced *C*. *albicans* infection. In vitro experiments confirmed that Nrf2 negatively regulates NLRP3 and inhibits the secretion of pro‐inflammatory cytokines. In combination with clinical data, smoking may suppress oral mucosa innate immunity and increase the possibility of infection by pathogenic microorganisms such as *C*. *albicans* in OLK. Studies have shown that ROS regulate both Nrf2 pathway and NLRP3 inflammasome, which may influence the antagonistic relationship between Nrf2 and NLRP3.^41^ In addition, Nrf2 inhibits NLRP3 inflammasome activation in macrophages by inducing NQO‐1 expression.^42^ Therefore, the specific mechanism through which this occurs is worthy of further exploration.

This study had some limitations. Most markers in rats were determined in the serum and are representative of systemic effects and not niche‐specific to the oral cavity. Therefore, saliva would have been a more appropriate sample for this study. Unfortunately, salivary production is very low in rodents, and it is challenging to collect enough saliva for assays. Furthermore, although we have confirmed the role of Nrf2 in smoking‐induced *C*. *albicans* infection in cell culture experiments, we have not verified it in in vivo experiments, such as those using Nrf2 knockout animal models. We hope to address this shortcoming in the future.

## 5 CONCLUSIONS

In conclusion, the negative regulatory effect of Nrf2 on NLRP3 is involved in smoking‐induced oral mucosa immunosuppression caused by smoking, thus increasing oral mucosal susceptibility to *C*. *albicans*. Nrf2, which is linked to oxidative stress and inflammation, is considered to be a key regulator and a potential therapeutic target for smoking and *C*. *albicans*‐related oral diseases, such as OLK.

## CONFLICT OF INTEREST

None declared.

## AUTHOR CONTRIBUTIONS

**Pei Ye:** Data curation (equal); Formal analysis (lead); Methodology (lead); Software (equal); Writing‐original draft (lead); Writing‐review & editing (lead). **Wei Chen:** Data curation (equal); Investigation (equal); Methodology (equal); Software (equal). **Fan Huang:** Investigation (equal). **Qin Liu:** Investigation (equal). **Ya‐Nan Zhu:** Funding acquisition (supporting); Investigation (equal). **Xiang Wang:** Funding acquisition (equal); Project administration (equal); Supervision (lead); Writing‐review & editing (equal). **Xiao‐Dong Han:** Project administration (equal); Validation (equal); Writing‐review & editing (equal). **Wen‐Mei Wang:** Conceptualization (lead); Funding acquisition (lead); Validation (equal).

## Supporting information

Supplementary Material

## Data Availability

Data will be made available on request to the corresponding author.
