# Supplementary material for: Smoking increases oral mucosa susceptibility to Candida albicans infection via the Nrf2 pathway: In vitro and animal studies
Source: J Cell Mol Med. 2021 Jun 21;25(16):7948–60. doi: 10.1111/jcmm.16724 (PMC8358876; doi:10.1111/jcmm.16724)
Supplement: Supplementary file 1 — Supplementary Material [file JCMM-25-7948-s001.docx]

Fig. S1. SFN and LV-Nrf2 could effectively regulate the expression of Nrf2. (A) The mRNA of Nrf2 was measured in transfected Leuk1 cells by Q-PCR. (B) The protein expression level of Nrf2 in transfected Leuk1 cells was evaluated by immunofluorescence analysis. (original magnification: × 400 for staining, scale bar: 30 μm). (C) The protein expression level of the Nrf2 pathway was evaluated by western blotting. Cells were treated with different concentrations of SFN. (D) Densitometry of western blots. GAPDH was used as a loading control. Graphs show the densitometric analysis (protein/GAPDH) of each band. *P < 0.05, **P < 0.01, compared with control. SFN: sulforaphane.


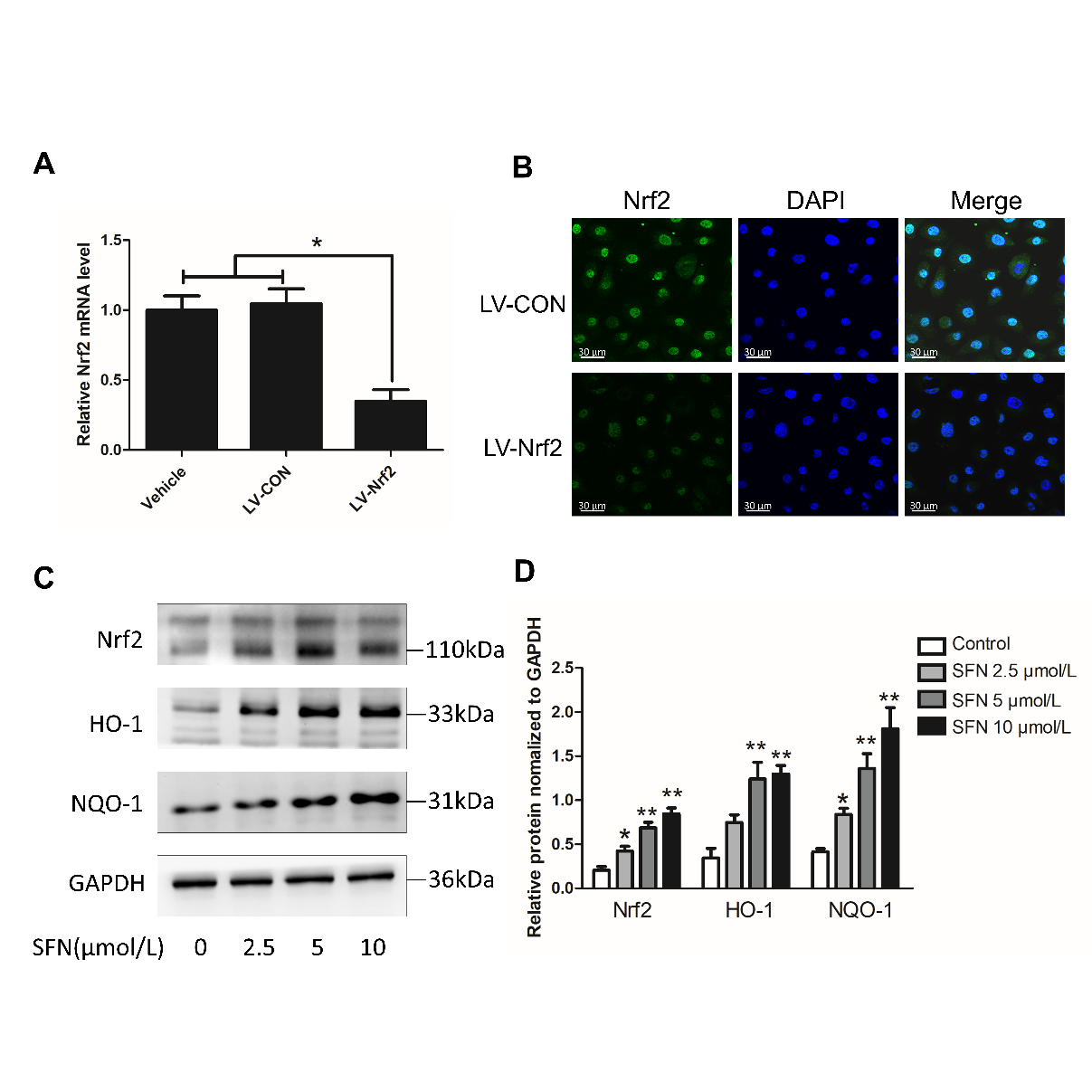


Supplement Table 1. Primers used for Q-PCR (h and r indicate human and rat species, respectively)

| Gene | Primer Sequences (forward, reverse) |
| --- | --- |
| Nrf2(r) | Forward：ATTTGATTGACATCCTTTGGAGGCA  Reverse：GGAATGTGGGCAACCTGGGAGTA |
| HO-1(r) | Forward：GGTGACAGAAGAGGCTAAGACCG  Reverse：CAACACTGCATTTACATGGCATAA |
| NQO-1(r) | Forward：CAAGTCCATTCCAGCCGACAACC  Reverse：CACAGCCGTGGCAGAACTATCCA |
| GAPDH(r) | Forward：GAACATCATCCCTGCATCCA  Reverse：CCAGTGAGCTTCCCGTTCA |
| Nrf2 (h) | Forward：TACTCCCAGGTTGCCCACA  Reverse：CATCTACAAACGGGAATGTCTGC |
| HO-1 (h) | Forward：CAACAAAGTGCAAGATTCTG  Reverse：TGCATTCACATGGCATAAG |
| NQO-1 (h) | Forward：GGATTGGACCGAGCTGGAA  Reverse：AATTGCAGTGAAGATGAAGGCAAC |
| GAPDH(h) | Forward：GGAGCGAGATCCCTCCAAAAT  Reverse：GGCTGTTGTCATACTTCTCATGG |
